# Construction of Two mCherry Plasmids (pXG-mCherry) for Transgenic* Leishmania*: Valuable Tools for Future Molecular Analysis

**DOI:** 10.1155/2017/1964531

**Published:** 2017-02-13

**Authors:** Andrés Vacas, Conor Sugden, Óscar Velasco-Rodriguez, Miriam Algarabel-Olona, José Peña-Guerrero, Esther Larrea, Celia Fernández-Rubio, Paul A. Nguewa

**Affiliations:** ^1^Instituto de Salud Tropical (ISTUN), University of Navarra, Pamplona, Spain; ^2^IdiSNA, Navarra Institute for Health Research, Pamplona, Spain; ^3^Department of Microbiology and Parasitology, University of Navarra, Pamplona, Spain

## Abstract

*Leishmania* is the causative agent of leishmaniasis, a neglected tropical disease that affects more than 12 million people around the world. Current treatments are toxic and poorly effective due to the acquisition of resistance within* Leishmania* populations. Thus, the pursuit for new antileishmanial drugs is a priority. The available methods for drug screening based on colorimetric assays using vital dyes are time-consuming. Currently, the use of fluorescent reporter proteins is replacing the use of viability indicator dyes. We have constructed two plasmids expressing the red fluorescent protein mCherry with multiple cloning sites (MCS), adequate for N- and C-terminal fusion protein constructs. Our results also show that the improved pXG-mCherry plasmid can be employed for drug screening* in vitro*. The use of the red fluorescent protein, mCherry, is an easier tool for numerous assays, not only to test pharmacological compounds, but also to determine the subcellular localization of proteins.

## 1. Introduction

Leishmaniasis is a neglected tropical disease caused by protozoans of the genus* Leishmania*. According to the World Health Organization (WHO), 350 million people are at risk of infection as no commercial vaccines are available for its prevention [1]. The WHO emphasizes the urgent need for developing new drug treatments, vaccines, and more specific and sensitive diagnostic methods.

Since the recognition of* Leishmania *spp. as the causative agent of leishmaniasis, generic pentavalent antimonials have been the essential chemotherapy agents against this disease. Pentostam® and Glucantime® are the branded alternatives to generic antimonials treatment. Other treatments such as amphotericin B and miltefosine offer a higher efficacy but a costlier option than pentavalent antimonials. Antipathogens like paromomycin and pentamidine show some benefit in the treatment of leishmaniasis, especially when used in conjunction with other drugs. But all these pharmacological compounds raise severe side effects without the confidence of complete healing and currently, and various* Leishmania* strains have developed resistance against these drugs [2].

The analysis of the potential leishmanicidal activity of new drugs may be performed by colorimetric assays that require the use of dyes with limitations such as being time-consuming. Some dyes currently applied in research are, for example, MTT (3-(4,5-dimethylthiazol-2-yl)-2,5-diphenyltetrazolium bromide) which measures cell metabolic activity or resazurin (7-hydroxy-3*H*-phenoxazin-3-one 10-oxide), a blue dye used as an indicator for cell viability [3–6], based on oxidation-reduction process. Limitation of reporter dyes has led to the use of genetically modified* Leishmania* parasites for bioluminescent and fluorescent drug screening that does not require the use of the aforementioned dyes.

Luciferase-expressing* Leishmania* parasites have shown to be effective for* in vitro* and* in vivo* studies during experimental infections [7, 8]. However, the luminescence assays need the addition of expensive substrates such as coelenterazine or luciferin, to detect the light emitted by the transgenic organisms. This makes fluorescent* Leishmania* more useful for* in vitro* tests. Actually, fluorescent reporter proteins offer a very stable signal over time that does not need specific substrates and allows the identification of single parasites in tissue studies [9, 10]. Recent advances in parasites expressing reporter gene constructs have proven to be a rapid method, with a high throughput/output for drug screening [11]. It is well known that the discovery of new potentially effective chemical compounds against this disease remains a priority.

In addition, fluorescent protein plasmid constructions can offer the advantage of adding gene sequences inside the same reading frame for fusion protein attainment, being useful tools to determine subcellular localization of proteins of interest [12].

mCherry protein derived from* Discosoma striata *displays higher photostability and tissue penetration than GFP (Green Fluorescent Protein), within the range of 610 nm and 587 nm excitation wavelength [13]. This red fluorescent protein has proven to be a great tool for* in vitro* and* in vivo* assays as well as drug screening [2, 9, 14]. By using the* Leishmania* expression plasmid pXG, we have developed two new vectors expressing mCherry for C- and N-terminal fusion protein in* Leishmania *spp. We also improved the pXG-HYG plasmid by adding new restriction enzyme sites within its cloning cage, suitable for easier and better fusion protein constructs.

## 2. Material and Methods

### 2.1. Parasites and Animals

*L. major* promastigotes were grown at 26°C in M199 medium (without phenol red; Sigma-Aldrich, St. Louis, USA) supplemented with 25 mM HEPES (pH 7.2; Sigma-Aldrich), 0.1 mM adenine (Sigma-Aldrich), 0.0005% (wt/vol) hemin (Sigma-Aldrich), 2 mg/mL biopterin (Sigma-Aldrich), 0.0001% (wt/vol) biotin (Sigma-Aldrich), 10% (vol/vol) heat-inactivated fetal bovine serum (Gibco Laboratories, Grand Islands, USA), and 40 *μ*g/mL gentamycin (Sigma-Aldrich).

Female BALB/c mice were purchased from Harlan Interfauna Ibérica S.A. (Barcelona, Spain). The Animal Care Ethics Commission of the University of Navarra approved all the procedures involving animals.

### 2.2. pXG-mCherry Molecular Constructs

For the construction of novel vectors, the 711-bp mCherry coding region was amplified by PCR from pTREX-mCherry. For vector pXG-mCherry12 (designed for creating N-terminal fusion proteins), we used Fw primer CH01 with NotI and XbaI sequence and the Rv primer CH02 with BamHI (Table 1), whereas for pXG-mCherry34 vector (designed for creating C-terminal fusion proteins) Rv primer CH03 with NotI and Fw primer CH04 with BamHI and XbaI were used (Table 1).

PCR products were then ligated into pCR®2.1-TOPO® expression vector (ThermoFisher Scientific, Rockville, USA) following the manufacturer's instructions. The ligation products were used to transform DH5*α Escherichia coli *bacteria. Positive colonies were selected by resistance to kanamycin and the nucleotide sequence of* mCherry*; also its direction was confirmed by PCR.

**Figure 1:**
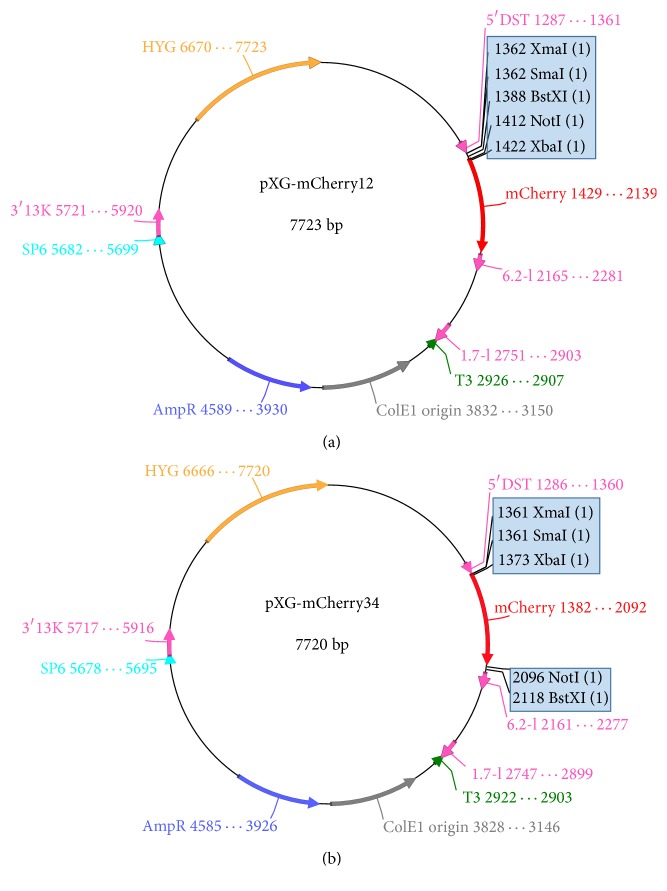
Schematic representation of pXG-mCherry plasmid constructs. (a) pXG-mCherry12. (b) pXG-mCherry34. HYG, hygromycin B phosphotransferase gene. Blue boxes depict the polylinker sequences (MCS: multiple cloning site). Vector map representation was implemented using ApE v2.0.48 [15].

*mCherry* gene sequence was then extracted, digested using BamHI, and ligated into the pXG-HYG* Leishmania* vector. As a result, two novel vectors were obtained, one designated as pXG-mCherry12 for creating N-terminal fusion proteins (Figure 1(a)) and the other named pXG-mCherry34 for creating C-terminal fusion proteins (Figure 1(b)). Moreover, direction and sequence of mCherry were confirmed by PCR.

pXG-mCherry12, pXG-mCherry34, and pXG-HYG were transfected into* L. major* log phase parasites by the electroporation method, using a Bio-Rad Gene Pulser apparatus as previously described [16]. Transfected parasites colonies were selected from M199 agar plates in the presence of hygromycin B Gold (InvivoGen Europe, Toulouse, France) at a final concentration of 250 *μ*g/mL and maintained in M199 with 150 *μ*g/mL hygromycin B. Growth curve analysis was performed with all transgenic colonies. For further* in vitro* studies, pXG-mCherry12 parasites were mainly used.

### 2.3. Fluorescence Microscopy

*mCherry* expression was then tested. pXG-mCherry12 promastigotes were centrifuged and stained with 4′,6-diamidino-2-phenylindole (DAPI I; Abbott-Vysis, Madrid, Spain). Then, cells were washed twice with phosphate-buffered saline (PBS; ThermoFisher Scientific, Rockville, USA) and fixed with 1% formaldehyde.

To observe the fluorescence from pXG-mCherry12* Leishmania* amastigotes, murine peritoneal macrophages from 4- to 6-week-old BALB/c mice were used for the study. Animals were inoculated with 2 mL sterile thioglycolate (3%) broth (BD Difco) 72 hours prior to peritoneal cavity lavage with 5 mL of cold RPMI medium. The macrophages were then removed with a syringe as previously described by Neal and Croft (1984) [17] and seeded in 8-well culture chamber slides (LabTek; BD Bioscience, Erembodegem, Belgium) at a density of 2 × 10^4^ cells per well in RPMI medium and allowed to adhere overnight at 37°C in a 5% CO_2_ incubator. Metacyclic pXG-mCherry12 promastigotes were isolated from stationary cultures by negative selection as described by Sacks et al. (1985) [18] using peanut agglutinin (PNA) and were used to infect macrophages at a ratio of 1/10 (macrophage/parasite). Plates were incubated for 24 hours under the same conditions and the wells were washed twice with PBS to remove nonintracellular parasites. Finally, cells slides were stained with 4′,6-diamidino-2-phenylindole (DAPI I; Abbott-Vysis, Madrid, Spain), fixed with 1% formaldehyde, and observed under a Nikon Eclipse 80i microscope.

### 2.4. Measurement of the Correlation between Fluorescence Intensity and Parasite Number

10^5^ pXG-mCherry12 metacyclic promastigotes isolated by negative selection (PNA method) were injected into the base of the tail of each mouse in a volume of 100 *μ*L of phosphate-buffered saline (PBS; ThermoFisher Scientific, Rockville, USA). In order to recover live parasites, four weeks after the infection, livers from the infected mice were homogenized in Schneider medium supplemented with 20% of FBS and incubated at 26°C to allow amastigotes to differentiate back to fluorescent promastigotes.

In order to assess the correlation between parasite number and fluorescence intensity, an increasing number of parasites (obtained before and after* in vivo* infection) were seeded in black 96-well plates with clear bottom and their fluorescence intensity was measured spectrofluorometrically. Three independent experiments were performed.

### 2.5. Leishmanicidal Assays

Exponential-phase pXG-mCherry12 parasites were seeded in black 96-well plates with clear bottom (200 *μ*L per well) with increasing concentrations of amphotericin B and miltefosine, diluted in M199 medium and maintained at 26°C. After 48 and 72 h of incubation, the half-maximal effective concentration (EC_50_) was measured by two different techniques: fluorimetric and colorimetric methods. Fluorescence was monitored at a 570-nm excitation wavelength and a 620-nm emission wavelength using a BMG FLUOstar Optima microplate reader (BMG LabTech, Ortenberg, Germany). On the other hand, for the colorimetric assay 3-[4,5-dimethylthiazol-2-yl]-2,5-diphenyl-tetrazolium bromide (MTT) (Sigma, St. Louis, MO, USA) was used as previously described [19]. MTT solutions were prepared at 5 mg/mL in PBS. After adding 20 *μ*L of MTT solution to each well, the plates were incubated during 4 h at 26°C. Subsequently, 80 *μ*L of dimethyl sulphoxide (DMSO, Panreac, Spain) was added to each well to dissolve formazan crystals. The optical density (OD) was measured using a Multiskan EX Microplate Photometer plate reader at 540 nm.

The EC_50_ was obtained by fitting a sigmoidal Emax model to dose-response curves. In both cases, promastigotes viability was evaluated based on a comparison with untreated control cells. The results were expressed as mean ± standard error (SEM) from three independent experiments.

### 2.6. Localization of a LPG1-mCherry Fusion Protein

*LPG1* (lipophosphoglycan) sequence was amplified from genomic* Leishmania major* DNA by PCR using primers LPG-Fw and LPG-Rv (Table 1). Then, PCR product was ligated into pCR2.1-TOPO expression vector and used to transform DH5*α E. coli *bacteria. The plasmid extracted from the recombinant bacteria was restriction enzyme digested with NotI. Gel purification corresponding to the* LPG1* sequence was subcloned into pXG-mCherry34 plasmid within the NotI restriction enzyme site.* LPG1* sequence and its direction inside pXG-mCherry34 were confirmed by PCR and sequencing. The obtained plasmid (pXG-mCherry34-LPG1) was used to electroporate* Leishmania major* cells following the aforementioned protocols of parasites transfection and colony selection. Finally, mCherry-LPG1 protein expression and localization within transgenic parasites were confirmed by fluorescence microscopy.

### 2.7. Statistical Analysis

Statistical analyses were executed with GraphPad Prism 6.0h (GraphPad Software Inc., San Diego, CA, USA) [20]. Two group comparisons were performed by employing unpaired, two tailed Student's* t-*test.* P *values > 0.05 were considered nonsignificant. Data were represented either as mean ± SEM or as mean ± SD.

## 3. Results

### 3.1. Construction of Two pXG-mCherry Vectors Containing Novel Multiple Cloning Sites (MCS)

A DNA region containing the coding sequence for mCherry with different MCS was inserted into the* Leishmania* expression vector pXG, generating two pXG-mCherry vectors. Both constructs, called pXG-mCherry12 (7723 bp) and pXG-mCherry34 (7720 bp), also harboured the hygromycin resistance cassette (Figure 1). As observed in Figure 1(a), pXG-mCherry12 presented at the N-terminal of the fluorescent protein the following restriction enzyme sites: XmaI, SmaI, BstXI, and NotI, whilst pXG-mCherry34 exhibited XmaI, SmaI, and XbaI at N-terminal and NotI and BstXI at C-terminal of mCherry (Figure 1(b)). By PCR, pXG-mCherry12 and pXG-mCherry34 sequences were analysed and confirmed.

### 3.2. Generation of Two Fluorescent* L. major* Strains Harbouring pXG-mCherry12 and pXG-mCherry34, Respectively

**Figure 2:**
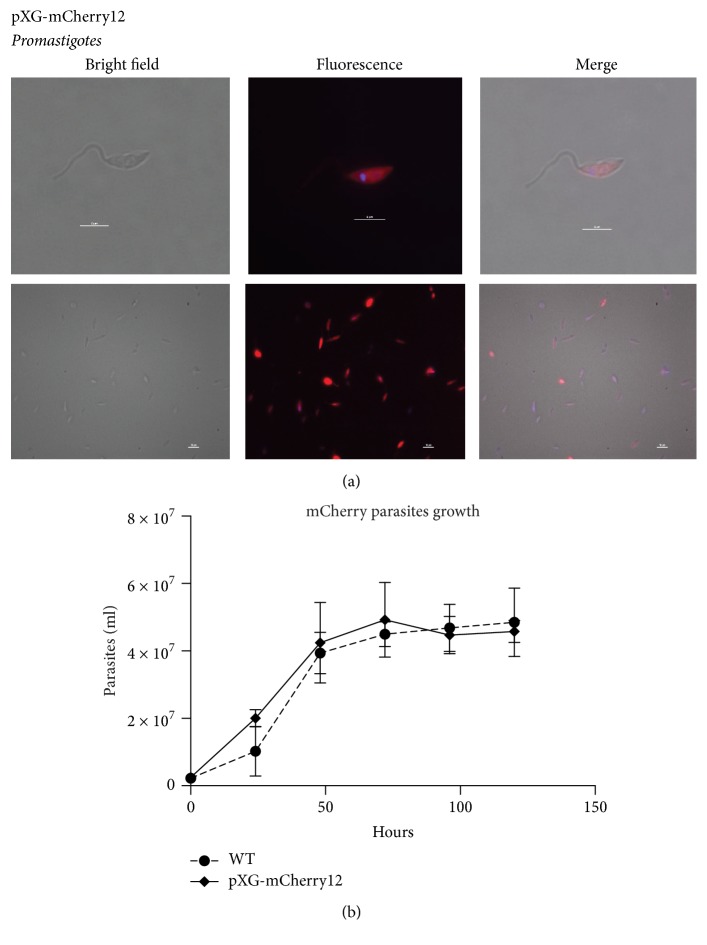
*L. major* pXG-mCherry12 parasites stably expressing the red fluorescent protein, upholding the same growth rate as the WT strain. (a) Fluorescent detection of red fluorescent parasites by confocal microscopy. Top lane, single parasite acquisition (100x objective). Bottom lane, more parasites per field (40x objective). Microscopy images were acquired with a Nikon Eclipse E800 confocal microscope. (b) Wild type strain and pXG-mCherry12 transfected parasites growth curve. Growth was measured during 6 days for WT (filled circle “●”) and pXG-mCherry12 (filled diamond “◆”) transfected parasites cultures. Data represents the mean (± SD) of three independent experiments.

After introducing pXG-mCherry12 into* L. major* by electroporation, various mutants were examined via fluorescent microscopy for mCherry expression. A notable red color could be observed throughout most of pXG-mCherry12 living parasites (Figure 2(a)). Additionally, mCherry fluorescence was assessed by flow cytometry (Supplementary Figure S2 in Supplementary Material available online at https://doi.org/10.1155/2017/1964531). The generation of fluorescent* L. major* strains was therefore confirmed.

With these data, we decided to assess the growth rate of pXG-mCherry12 clonal transfectants. As the growth curves illustrated, the rates of proliferation did not differ from those of the wild type (Figure 2(b)). In addition, the corresponding analysis showed no significant differences (Figure 2(b)).

### 3.3. mCherry Fluorescent* L. major *Parasites Exhibit Similar Drug Sensitivity Compared to Wild Type Cells

**Figure 3:**
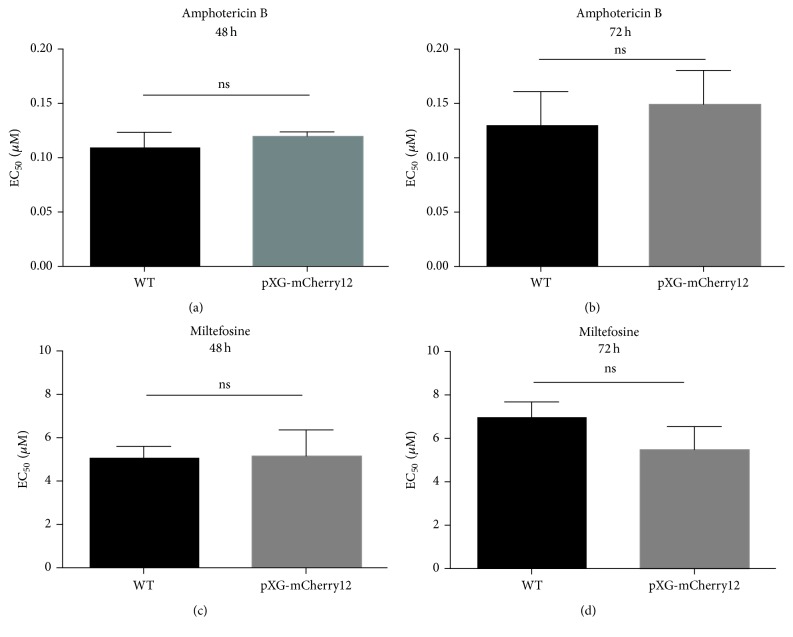
pXG-mCherry12 transfected parasites display similar EC_50_ values against both reference drugs: miltefosine and amphotericin B. (a and b) Amphotericin B EC_50_ values after 48 and 72 h exposure. (c and d) Miltefosine EC_50_ values after 48 and 72 h exposure. Bars indicate the mean (± SEM) from three independent experiments.

In order to analyse drug sensitivity, further experiments were carried out with our mutants and wild type cells using the classical MTT method. To assess this goal, current drugs used in the treatment of human leishmaniasis (miltefosine and amphotericin B) were evaluated in wild type and pXG-mCherry12* Leishmania* at different concentrations during 48 and 72 h. The EC_50_ values were then calculated. No differences between both groups were detected (Figure 3) thus reinforcing the aforementioned similarity of our clones with respect to controls.

### 3.4. Intracellular Forms Maintain mCherry Expression: Fluorescence Intensity Correlates with Parasite Number

**Figure 4:**
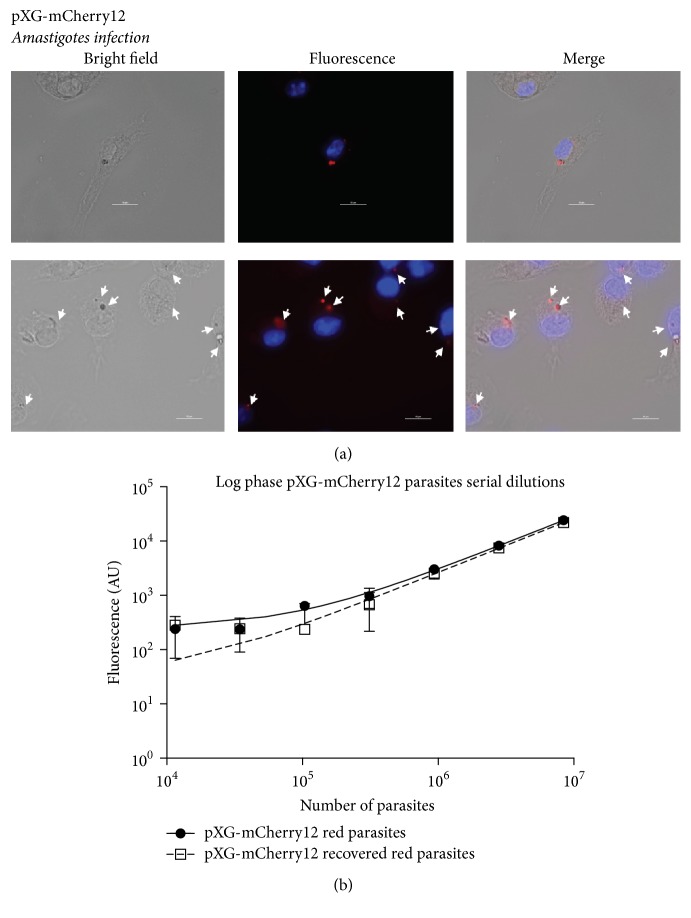
pXG-mCherry12 transfected parasites preserve their fluorescence during* in vitro* and after* in vivo* infections. (a) Murine peritoneal macrophages infected with red fluorescent parasites. Solid white arrows indicate fluorescent amastigotes. Microscopy images were acquired with a Nikon Eclipse E800 confocal microscope. (b) Fluorescence emitted from pXG-mCherry12 promastigotes recovered after* in vivo* infection. 96-well plates were read with a FLUOstar Optima plate reader using 570-nm excitation and 620-nm absorbance filters.

pXG-mCherry12 red fluorescent parasites were obtained from livers of BALB/c infected mice. 20% FBS Schneider's medium allowed the amastigote forms to differentiate back to fluorescent promastigotes. Metacyclic parasites, purified by PNA negative selection, were then used to infect murine peritoneal macrophages.  Figure 4(a) showed that infected macrophages harboured fluorescent amastigotes. Fluorescence from amastigotes was also quantified spectrofluorometrically (Supplementary Fig. S3). Supplementary Figure S3 showed additional evidence of the fluorescence emission from pXG-mCherry12 amastigotes after* in vitro* BMM (Bone Marrow-Derived Macrophages) infections. Interestingly, an increase of the infection ratio (macrophage : amastigotes = 1 : 12, 1 : 25, 1 : 50, and 1 : 100) also produced a higher fluorescence intensity. Therefore, mCherry-expressing transgenic cells maintained fluorescence after* in vivo* infections and in the intracellular form of our clones (Figure 4(a) and Supplementary Fig. S3).

Fluorescence evaluation from increasing number of parasites was used in order to assess the correlation between parasite quantity and fluorescence intensity. The experiment was performed with the parasites obtained before and after the* in vivo* infection to determine any fluorescence alteration. No fluorescence reduction was detected after the* in vivo* infection. As observed in Figure 4(b), after spectrofluorometric measurements, a clear correlation between absolute fluorescence intensity and the number of parasites (pXG-mCherry12 recovered parasites) was detected with a very significant correlation coefficient (*R*^2^ = 0.9997) (Figure 4(b)). Interestingly, a similar trend (*R*^2^ = 0.9999) had been previously observed in pXG-mCherry12 parasites versus fluorescence intensity (Figure 4(b)). Furthermore, the copy number of the plasmid was quantified in both samples (pXG-mCherry12 parasites and pXG-mCherry12 recovered parasites) and no difference was observed between both (~80 copies/cell; Supplementary Fig. S1). These data indicated a linear relationship between parasite number and the quantified fluorescence.

### 3.5. Comparison of the Novel Fluorimetric Assay and MTT Technique for the* In Vitro* Evaluation of Cell Viability (EC_50_)

**Figure 5:**
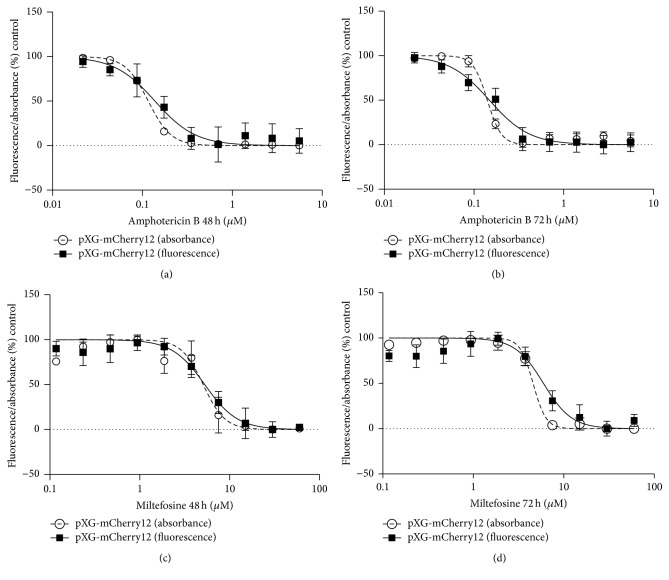
mCherry fluorescence is a valuable tool for measuring cell viability. Two pharmacological compounds were tested: amphotericin B (a and b) and miltefosine (c and d). Nonlinear regression curve fit for MTT (absorbance) and mCherry (fluorescence) is shown. Measurements were performed after 48 and 72 h for each drug. Plots represent the mean (± SD) of three independent experiments.

Absolute fluorescence emitted by cells was plotted versus concentrations of miltefosine and amphotericin B (Figure 5). In addition, MTT technique was also performed to evaluate growth inhibition. The corresponding dose-response curves were therefore compared (Figure 5).

After 48 h exposure to drugs, the following EC_50_ values were obtained (Table 2): 0.1368 ± 0.0121 *μ*M (fluorimetric assay) versus 0.12 ± 0.0040 *μ*M (MTT) for amphotericin B and 5.58 ± 1.022 *μ*M (fluorimetric assay) versus 5.16 ± 1.21 *μ*M (MTT) for miltefosine. Meanwhile after 72 h of treatment (Table 2), the EC_50_ values measured by fluorimetric assay and MTT were 0.13 ± 0.021 *μ*M and 0.15 ± 0.031 *μ*M (amphotericin B), respectively, and 6.32 ± 0.63 *μ*M and 5.48 ± 1.063 *μ*M (miltefosine), respectively.

The EC_50_ values obtained from both drugs, acquired by fluorimetric and colorimetric methods, are compared in Table 2. The statistical analysis revealed that there were no differences between both methods. All these data showed that the fluorimetric assay using this novel pXG-mCherry* Leishmania* is a useful tool for* in vitro* drug screening.

### 3.6. Application of Our Constructs to Localize Proteins within* L. major*

**Figure 6:**
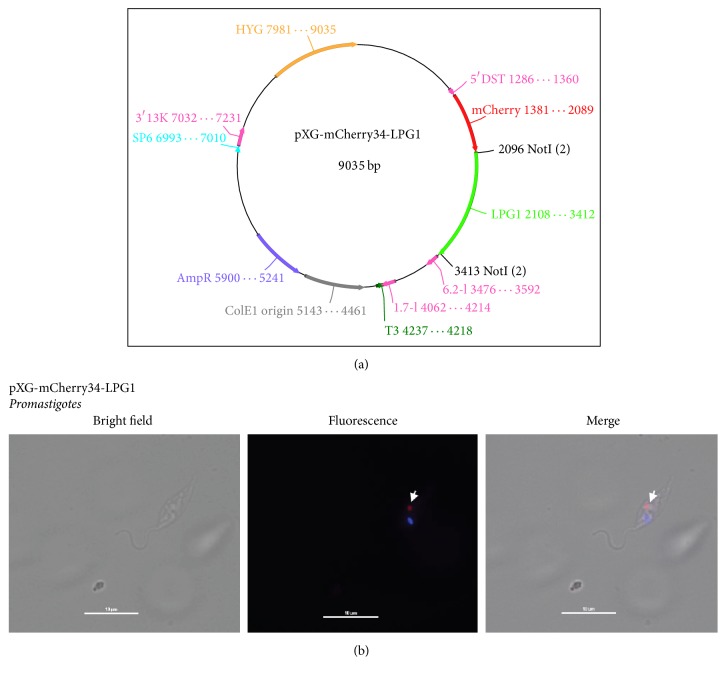
pXG-mCherry plasmids allow the generation of fusion proteins. (a) pXG-mCherry34-LPG1 construct, developed to assess protein localization. (b) Localization of mCherry-LPG1 fusion protein. Golgi apparatus where mCherry-LPG1 expression is observed (solid white arrow). Microscopy images were acquired with a Nikon Eclipse E800 confocal microscope.

One of the major applications of a fluorescent protein like mCherry is its use as a marker for cellular localization of fusion proteins. To assess this in* Leishmania*, we performed experiments allowing the fusion of mCherry protein to the N-terminus of LPG, yielding the construct pXG-mCherry34-LPG1 (Figure 6(a)). This novel vector and pXG-mCherry34 were then transfected into* L. major* promastigotes. pXG-mCherry34-expressing* L. major *showed a fluorescence pattern (not shown), identical to that previously described for pXG-mCherry12 (Figure 2). Moreover, mCherry-LPG1 transfectants exhibited localization of mCherry to a region situated in the Golgi apparatus, adjacent to the DAPI-stained kinetoplast (Figure 6(b)). This LPG1 (mCherry) fluorescence pattern was clearly similar to that obtained by other authors [12, 21, 22]. Therefore, the novel pXG-mCherry constructs are useful tools for the localization of protein of interest.

## 4. Discussion

Although the procyclic form of* Leishmania* is neither the infective nor the intracellular stage, its implementation as a low-cost, easy, and rapid assay may be valuable in analysing new drug candidates. The proposed approach is reliable and inexpensive.

We generated two novel vectors (pXG-mCherry12 and pXG-mCherry34) that harbour polylinker sequences (multiple cloning site, MCS) allowing us to easily introduce target genes. In addition, mCherry generates fluorescence emission in the cell. A further use of mCherry as well as other fluorescent reporter genes (GFP and RFP) has largely facilitated the screening and testing of potential drugs against parasites [2, 14, 23–25].

In fact, our results also demonstrated that mCherry-expressing parasites are a valid tool to calculate* in vitro* the EC_50_ values of different leishmanicidal drugs. Such use is supported by interesting data obtained in this paper. Firstly, the growth curve of mCherry clones was identical to that of the WT parasites. Secondly, EC_50_ values from fluorescence assays showed no differences when compared to the values obtained through MTT staining. And finally, since pXG-mCherry12 and pXG-mCherry34 measurable red fluorescence significantly correlates with cell number, these novel vectors offer a high sensitivity and specific tool to measure drug efficacy instead of using methods based on metabolic activity such as MTT, more tedious and time-consuming. Interestingly, our results are in accordance with data obtained by MTT method.

Although this is one of the first steps for validating new drugs, pXG-mCherry parasites may represent, particularly in laboratories with limited resources, a good and inexpensive alternative for drug screening against leishmaniasis. However, the assessment of drug activity in amastigotes remains critical for selecting new leishmanicidal agents. Therefore, red fluorescence intensity of amastigotes may be a useful technique to achieve this goal. As we demonstrated,* Leishmania major *cells transfected with our novel mCherry vectors maintained red fluorescence during the amastigote stage. This outcome can also be exploited as a suitable way to determine parasite burden.

Furthermore, the introduction of multiple cloning sites (MCS) in our new constructs facilitates the easy insertion of genes of interest and allows the detection of their subcellular locations. In this work, we used* LPG1* gene to validate our method. Our results confirmed LPG1 localization in the Golgi apparatus as previously described by other authors [12, 21, 22].

This paper does not claim demonstrating that episomal expression is better than that obtained after integration at the level of the ribosomal locus. One of our aims was to generate a novel and useful tool especially for protein localization. We found that parasites stably harboured the episomal vector (~80 copies/cell) and throughout our study the corresponding fluorescence intensities were similar. Moreover, the episomal expression of mCherry reporter gene remained stable during several weeks allowing us to perform numerous experiments.

However, since the fluorescence intensity of the mCherry-expressing parasite was decreasing with time in* Leishmania* cells grown in the absence of hygromycin pressure (Supplementary Fig. S4 and S5), we propose that it may be interesting to obtain* Leishmania* cells stably expressing the* mCherry* gene from the parasite genome (integration at the level of the ribosomal locus). In our case, the integration into the genome may be useful but not necessary to achieve our main goals (protein localization).

When dealing with the episomal and/or integrative expression of genes, we need to keep in mind several data. Integrative reporter genes are valuable tools for extended growth assays, such as* in vivo* studies, since the expression of the protein of interest is independent of the selection marker. Nevertheless, our aim was mainly focused on* in vitro* activity tests in the promastigote forms. Episomal and stable expression of the luciferase reporter gene had been studied by Roy et al. (2000) [26]. Their work revealed that, for an equal number of parasites, luciferase activity was lower in LUC-gene integrated parasites than when the gene was part of an episome. However, the level of LUC-RNA expression in the cells presenting the integrated arrangement was close to the one derived from parasites harbouring the episomal vector [26]. It should be considered that the target gene within episomal plasmids cannot be interrupted or subjected to regulatory constrains which may occur after integration process. Finally, episomal constructs do not lead to the rearrangement or interruption of the cell genomic regions [27].

## 5. Conclusion

This paper proposes a rapid, easy, and reliable approach suitable not only to test* in vitro* new drugs and treatments against leishmaniasis, but also to study molecular biology features of the parasite. To our knowledge, this is the first report in which mCherry was inserted into pXG adding supplementary MCS. Finally, the novel vectors (pXG-mCherry) are remarkable tools to assess protein localization.

## Supplementary Material

Cultures populations harbouring episomal reporter displayed heterogeneous fluorescence intensity (Supplementary Figure S2A). A decrease in the median fluorescence intensity (MFI) of the parasites recovered from mouse tissue was observed (Supplementary Figure S2B). Globally, more than 85% of cell population showed mCherry red fluorescence.

## Figures and Tables

**Table 1:** Oligonucleotides used in this work.

Oligonucleotide	Sequence (5′ → 3′)
CH01 (Fw)	g aGC GGC CGC gaT CTA GAc **atg gtg agc aag ggc ga**
CH02 (Rv)	c tGG ATC Cct cga gct **tta ctt gta cag ctc gtc cat**
CH03 (Rv)	a tGC GGC CGC tcg agt **ctt gta cag ctc gtc cat gc**
CH04 (Fw)	g GGA TCC TCT AGA GCc **atg gtg agc aag ggc gag ga**
LPG1-Fw	at GCG GCC GCc acc **atg gcg cct cgt cgc tgg cat**
LPG1-Rv	ga GCG GCC GC **tta gct agg atc aac agc aaa**

*Note*. Uppercase letters represent added restriction enzyme sites. In bold is the *mCherry/LPG1* gene sequence.

**Table 2:** Drug activity profile of *L. major* cell lines. Promastigotes were grown, as described in Materials and Methods, for 48 and 72 hours at 26°C in the presence of increasing drug concentrations. The 50% effective concentrations (EC_50_) were measured using an MTT-based assay and a FLUOstar Optima fluorimeter for WT and pXG-mCherry12 parasites. Results are expressed as mean (± standard error [SEM]) from three independent experiments.

Parasite	EC_50_ (*μ*M) mean ± SEM							
	Amphotericin B (48 h)		Amphotericin B (72 h)		Miltefosine (48 h)		Miltefosine (72 h)	
	MTT	Fluorescence	MTT	Fluorescence	MTT	Fluorescence	MTT	Fluorescence
WT	0.11 ± 0.014		0.13 ± 0.031		5.075 ± 0.52		6.97 ± 0.71	
pXG-mCherry12	0.12 ± 0.0040	0.14 ± 0.012	0.15 ± 0.031	0.13 ± 0.021	5.16 ± 1.21	5.58 ± 1.022	5.48 ± 1.063	6.32 ± 0.63
