# Supplementary material for: Construction of Two mCherry Plasmids (pXG-mCherry) for Transgenic Leishmania: Valuable Tools for Future Molecular Analysis
Source: J Parasitol Res. 2017 Feb 13;2017:1964531. doi: 10.1155/2017/1964531 (PMC5327783; doi:10.1155/2017/1964531)
Supplement: Supplementary file 1 — Cultures populations harbouring episomal reporter displayed heterogeneous fluorescence intensity (Supplementary Figure S2A). A decrease in the median fluorescence intensity (MFI) of the parasites recovered from mouse tissue was observed (Supplementary Figure S2B). Globally, more than 85% of cell population showed mCherry red fluorescence. [file 1964531.f1.pdf]

SUPPLEMENTARY FILE

Construction of two mCherry plasmids (pXG-mCherry) for transgenic *Leishmania*: valuable tools for future molecular analysis

Andrés Vacas[1,2], Conor Sugden[1,2], Óscar Velasco-Rodriguez[1,2], Miriam Algarabel-Olona[1,2], José Peña-Guerrero[1,2], Esther Larrea[1], Celia Fernández-Rubio[1,2], Paul A. Nguewa[1,2#]

[1] *Instituto de Salud Tropical (ISTUN). University of Navarra, Pamplona, Spain; IdiSNA, Navarra Institute for Health Research*

[2] *Department of Microbiology and Parasitology, University of Navarra, Pamplona, Spain.*

# Corresponding author:

Paul Nguewa, PhD

E-mail: panguewa@unav.es

Molecular Parasitology, Therapeutic Targets and Drug Discovery. Instituto de Salud Tropical (ISTUN). Department of Microbiology and Parasitology. University of Navarra. Avda. Pio XII, 55. 31008 Pamplona, Spain.

Material and methods

pXG-mCherry12 copies quantification

Log-phase cultures of *L. major* WT, pXG-mCherry12 and pXG-mCherry12 recovered parasites, were counted using a Z1 Coulter counter (Beckman Coulter, Fullerton, CA, USA). Three samples containing 10^8 parasites from each culture were used for DNA extraction using a NucleoSpin® Tissue extraction kit (Macherey-Nagel, Germany) following manufacturer instructions. The standard curve was made from serial dilutions (1:10) of pXG-mCherry12 plasmid with a concentration of 391.9 ng/µl. The number of plasmid copies was calculated as previously described by Lee C. *et al*. (2005) [1]. 1µl of DNA from each sample were used in a 10 µl reaction mix with 0.2 µl of primers CHq-Fw (5'-cccgccgacatccccgacta) and CHq-Rv (5'-gggtcacggtcaccacgcc) (15µM), 5 µl concentration of iQ™ SYBR® Green Supermix (Bio-Rad, California) and 3.6 µl of autoclaved water. qPCR was executed with an Applied Biosystems 7500 Real-Time PCR System; results were analysed using manufacturer provided software.

Flow cytometry analysis

10^6 log-phase *L. major* WT, pXG-mCherry12 and pXG-mCherry12 recovered parasites, were fixed with a solution of 2% paraformaldehyde in PBS for 15 min at room temperature. Afterwards, cells were washed twice and re-suspended in a final volume of 500 µl of PBS.

Data acquisition was performed with a BD FACSaria III (Becton Dickinson) equipped with 488nm, 561 nm and 633 nm laser lines and BD FACSdiva Software

(Becton Dickinson). mCherry was excited with a 561nm laser line and captured with a 610/20 BP filter. FSC/SSC signals were acquired on linear amplification and fluorescence signals with logarithmic amplification. Event Rate was 150-200 events/second. Before acquisition quality control was done using CST beads according with manufacturer instructions.

Analyses were done on singlets, excluding debris on FSC/SSC and doublets with BD FACSdiva (Becton Dickinson), or FlowJo software (TreeStar). More than 20,000 events were analysed for each test. Negative controls were used to set the negative boundaries and establish the positive populations.

The Fluorescence intensities were reported in arbitrary units as MFI (Median Fluorescence Intensity), or in the whole population singlets. The percentages of mCherry+ cells on singlets were also reported.

BMM (Bone Marrow-derived Macrophages) isolation and amastigotes fluorescence quantification

Cells from femurs and tibias from BALB/c mice were differentiated into macrophages for 8 days using DMEM (Gibco Laboratories, Grand Islands, USA) supplemented with 5µg/ml penicillin, 100 U/ml streptomycin 10% fetal bovine serum and L-929-conditioned medium, following the procedure previously described by Van den Bossche *et al.* (2015) [2]. BMMs (Bone Marrow-derived Macrophages) were counted using a Neubauer chamber and re-suspended in DMEM medium. Then, 20,000 cells were seeded per well in black 96-well plates with clear bottom. 24 h after, PNA-isolated metacyclic pXG-mCherry12 parasites were added to each well at different concentrations to achieve four

infection ratios: 1:12, 1:25, 1:50 and 1:100. Infections were synchronized by centrifugation (330 xg, 3 min at 4 ºC) and plates were incubated for 24 h at 37ºC with 5% CO_2. Finally, plates were washed three times with warm PBS and fluorescence was quantified using a BMG FLUOstar Optima microplate reader as previously described.

Fluorescence quantification of Hygromycin-free medium (HFM) cultures

pXG-mCherry12 parasites were washed twice with Hygromycin-free complete M199 medium (without phenol-red) to remove Hygromycin (the selection antibiotic). Then, cultures were grown for 7 and 60 days at 26ºC in Hygromycin-Free Medium (HFM). After 60 days, a subculture was made and grown in medium supplemented with hygromycin. Fluorescence was measured spectrofluorometrically as previously described, at different conditions: a) 7 and 60 days in HFM; and b) 60 days in HFM and then in Hygromycin-supplemented medium).

Results

pXG-mCherry12 plasmid copies quantification and flow cytometry analysis

We have quantified the copy number of our clone by absolute qPCR using the standard curve method (R^2=0.9968). We analysed the copy number from two different samples already reported in the manuscript: **a)** the fluorescent cells obtained after colony selection from agar plates (**pXG-mCherry12 parasites**); **b)** pXG-mCherry12 parasites grown *in vitro*, PNA-selected and used to infect animals (*in vivo* assays), and the subsequent cells obtained from the culture derived from the *in vivo* infections (**pXG-mCherry12 recovered parasites**). Therefore, the recovered parasites were maintained 60 days in our Lab (compared to the pXG-mCherry12).

As shown in **Supplementary Figure S1B** and **Supplementary Table S1**, the number of copies from the recovered parasites was similar to that detected in pXG-mCherry12 parasites. These data demonstrated that there was no change in the number of copies of the plasmid after 60 days.

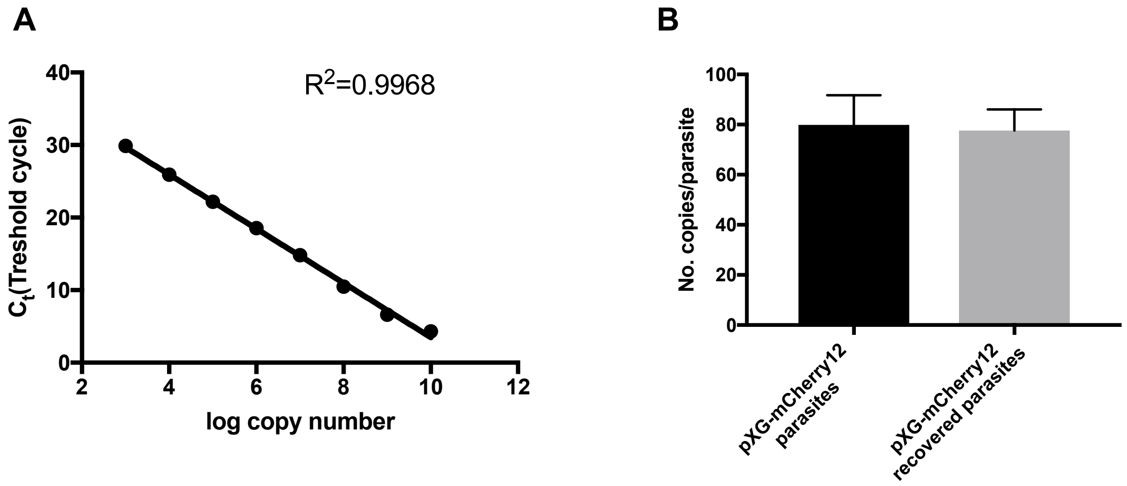

Figure S1. pXG-mCherry12 plasmid quantification by absolute qPCR. **A)** Plasmid copies standard curve (R^2=0.9968). **B)** Plasmid copies per parasite from each analysed sample (pXG-mCherry12 recovered parasites and pXG-mCherry12 parasites). Bars indicate the mean (± SEM).

Table S1. pXG-mCherry12 copies per parasite, measured by absolute qPCR (R^2=0.9968).

Culture	Copies[a] (copies/parasite)
**pXG-mCherry12 parasites**	79.90 ± 11.85
**pXG-mCherry12 recovered parasites**	77.66 ± 8.35

[a] Mean ± SEM (n=3)

We further analysed the fluorescence from these samples by Flow Cytometry technique (**Supplementary Figure S2A**). Cultures populations harbouring episomal reporter displayed heterogeneous fluorescence intensity (**Supplementary Figure S2A**) as already described by Calvo-Álvarez *et al.* (2012) [3]. A decrease in the median fluorescence intensity (MFI) of the parasites recovered from mouse tissue was observed (**Supplementary Figure S2B**). In addition, the percentage of mCherry+ cells did not vary in both populations (85.6% for pXG-mCherry12 parasites and 85.3% for pXG-mCherry12 recovered parasites).

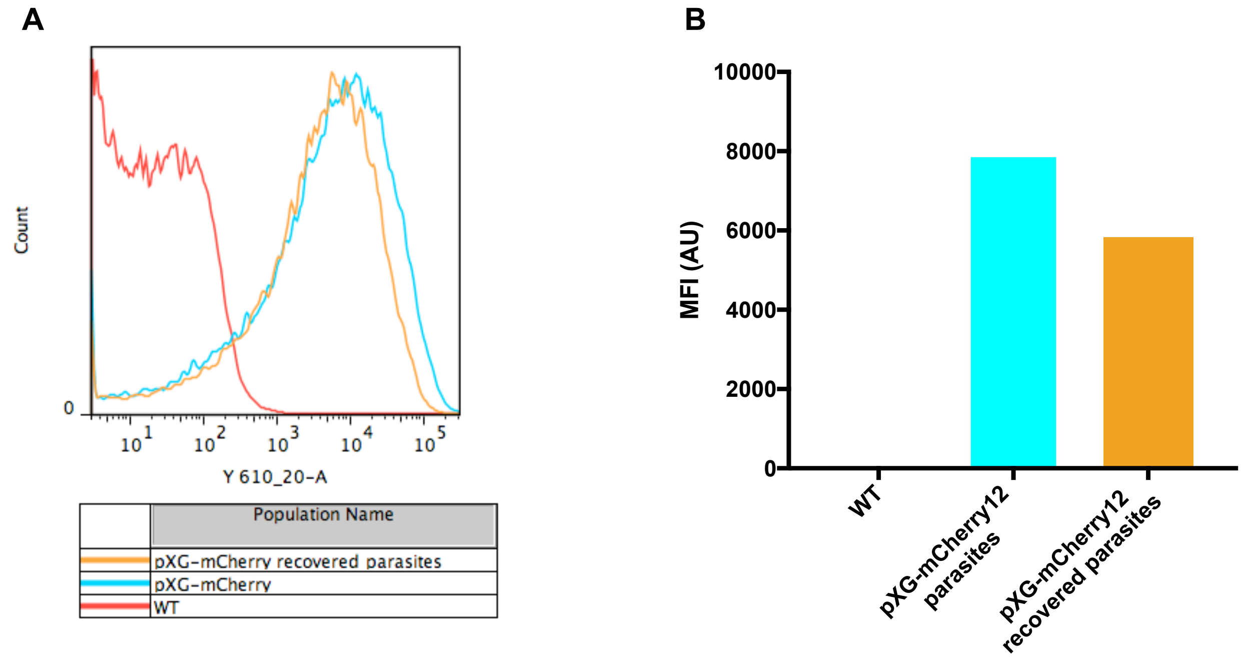

Figure S2. Analysis of the fluorescence by Flow Cytometry. A) Histogram plot representative of distribution of mCherry fluorescence levels in different populations. **B)** Median Fluorescence Intensity (MFI) of the WT, pXG-mCherry12 and pXG-mCherry12 recovered parasites.

Amastigotes fluorescence quantification

The red fluorescence of our plasmids was maintained **during *in vitro*** and after *in vivo* infections. As presented in **Figure 4A**, *Leishmania major* amastigotes harbouring pXG-mCherry12 (pXG-mCherry12 amastigotes) exhibited red-fluorescence inside murine peritoneal macrophages. On the other hand, **Figure S3** shows additional evidence of the fluorescence emission generated by pXG-mCherry12 amastigotes after *in vitro* BMM (Bone Marrow-derived Macrophages) infections. Interestingly, an increase of the infection ratio (macrophage:amastigotes= 1:12, 1:25, 1:50 and 1:100) also produced a higher fluorescence intensity.

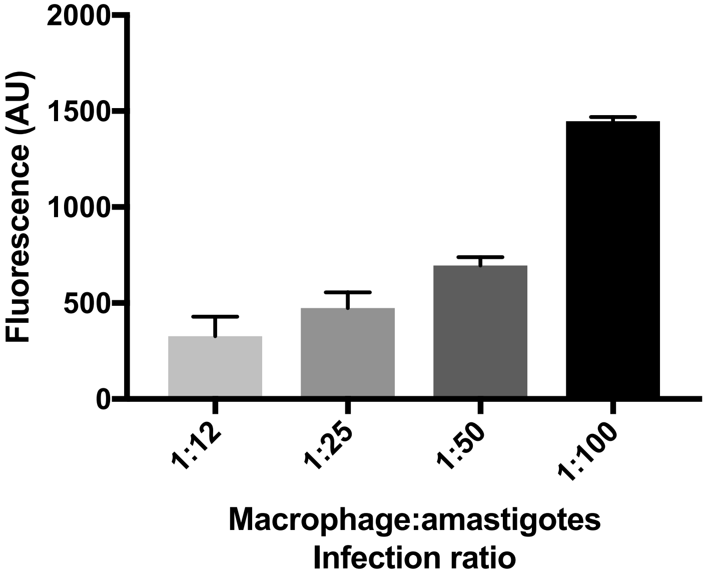

Figure S3. Mean fluorescence intensity emitted by amastigotes within macrophages. The fluorescence was expressed as arbitrary units (AU). 20,000 Bone Marrow-derived Macrophages (BMM) per well were seeded in a 96-well plate and infected with 4 different macrophage:amastigote ratios (1:12, 1:25, 1:50 and 1:100). Fluorescence was quantified using a BMG FLUOstar Optima microplate reader with 570/620 nm laser filters. Bars indicate the mean fluorescence (± SD).

mCherry fluorescence kinetics in Hygromycin free medium

We have conducted fluorescence intensity analysis. The values obtained under different conditions (Hygromycin-free medium, -HFM-; Hygromycin addition after 60 days in HFM) were compared to data previously measured from pXG-mCherry12 parasites grown in medium with hygromycin (**Supplementary Fig S4**). In fact, as shown in **Supplementary Figure S4**, a good correlation (R^2=0.9999) was observed between the number of pXG-mCherry12 parasites grown in medium with hygromycin and fluorescence intensity.

We then analysed red-fluorescence emission of pXG-mCherry12 parasites after both, short- (7 days) and long-term (60 days) culture in Hyg-free medium (HFM). Our data indicated that after 7 days cultured in HFM, no loss in fluorescence was appreciated when compared to controls (pXG-mCherry12 parasites grown in medium with hygromycin) (**Supplementary Figure S4A**).

We further studied fluorescence intensity when cultured in HFM for a longer time (60 days). As expected for antibiotic-dependent expression plasmid such as pXG, after 60 days of cultivation in HFM, parasites fluorescence reduction was detected (**Supplementary Figure S4A**). However, once in presence of Hygromycin, such cells were *de novo* able to emit fluorescence at the same level as controls (**Supplementary Figure S4B**). In fact, as observed in **Supplementary Figure S4B**, when parasites previously grown in HFM during 60 days were exposed to Hygromycin, the correlation between the number of these parasites and fluorescence intensity was high (R^2=0.9971) and the curve was similar to that of controls (**Supplementary Figure S4B**).

On the other hand, a similar trend was observed in promastigotes recovered from mouse tissue (pXG-mCherry12 recovered parasites) (**Supplementary Figure S4B**). This result (fluorescence recovery) is in accordance with the data reported in our manuscript (**Figure 4B**). Parasites obtained from *in vivo* infections recovered their initial fluorescence intensity after Hygromycin was added to the culture medium, the same way as parasites cultured in HFM recovered their fluorescence when Hygromycin was added (**Supplementary Figure S4B**).

Finally, the aforementioned observations were confirmed by microscopy (**Supplementary Figure S5**). As previously described, when pXG-mCherry12 parasites

were grown in Hygromycin-supplemented medium, red fluorescence emission was detected. Whereas, after pXG-mCherry12 parasites were cultured for 60 days in HFM, the fluorescence intensity decreased. On the contrary, after pXG-mCherry12 cells were grown in HFM (60 days) and then in Hygromycin-supplemented medium, the fluorescence intensity was recovered.

All together, these results suggest the stability of the plasmid within the parasite for a short period of time in HFM. Although such fluorescence intensity was reduced after long-term cultivation in HFM, our data demonstrated that it can be *de novo* recovered after adding hygromycin in the medium.

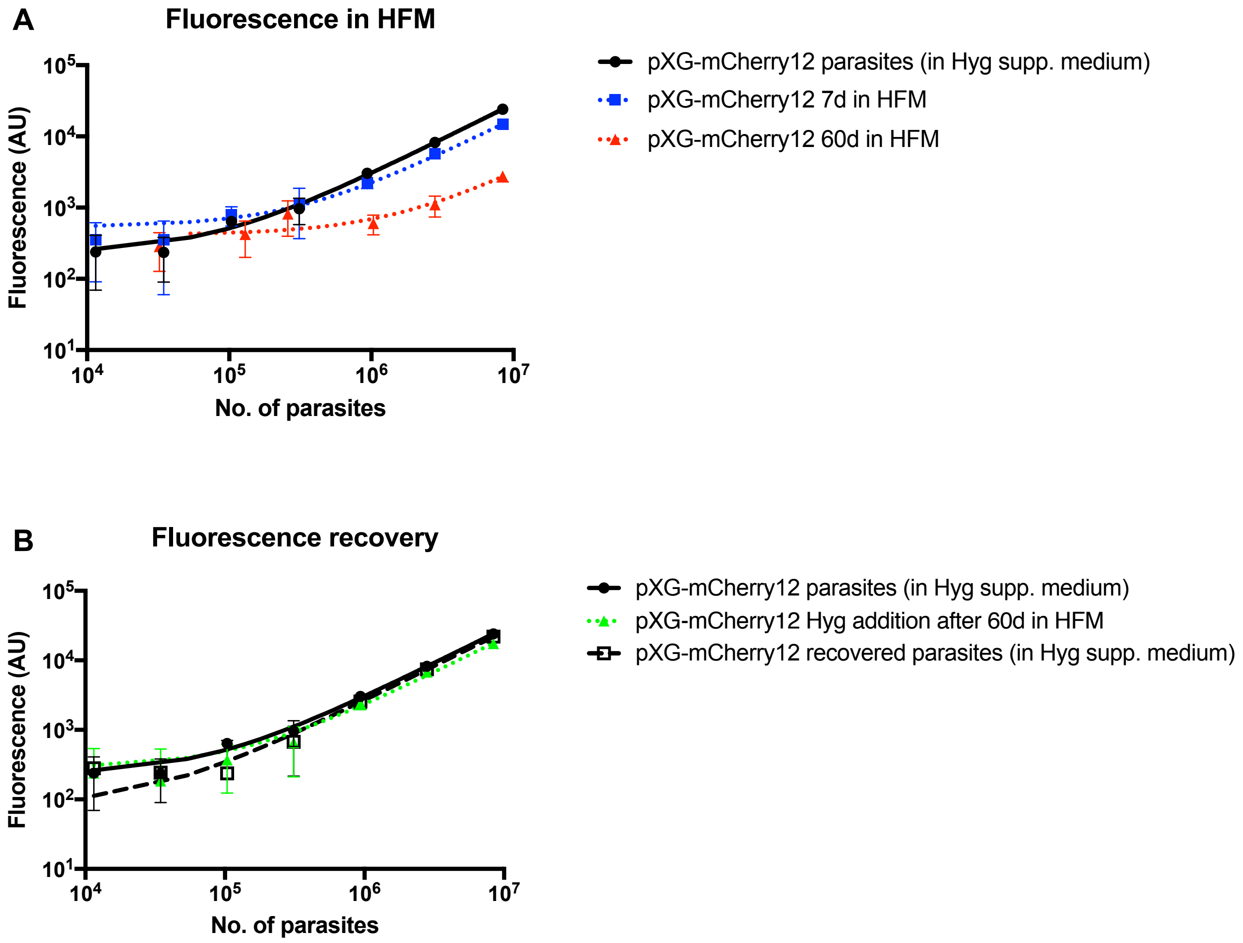

Figure S4. Fluorescence intensity of pXG-mCherry12 parasites was measured under different conditions (Hygromycin-free medium, -HFM- during 7 and 60 days; Hygromycin addition after 60 days in HFM) and compared to control (data previously measured from pXG-mCherry12 parasites grown in hygromycin medium, R^2=0.9999). **A)** Fluorescence emitted from pXG-mCherry12 promastigotes cultured for 7 or 60 days in HFM (R^2=0.9985 and 0.9639, respectively). **B)** Fluorescence when cultured in HFM for a longer time (60 days) and then in Hygromycin supplemented-medium (R^2=0.9971). A similar trend was observed in promastigotes recovered from mouse tissue (pXG-mCherry12 recovered parasites). Serial dilutions in 96-well plates were read with a FLUOstar Optima plate reader using 570-nm excitation and 620-nm absorbance filters.

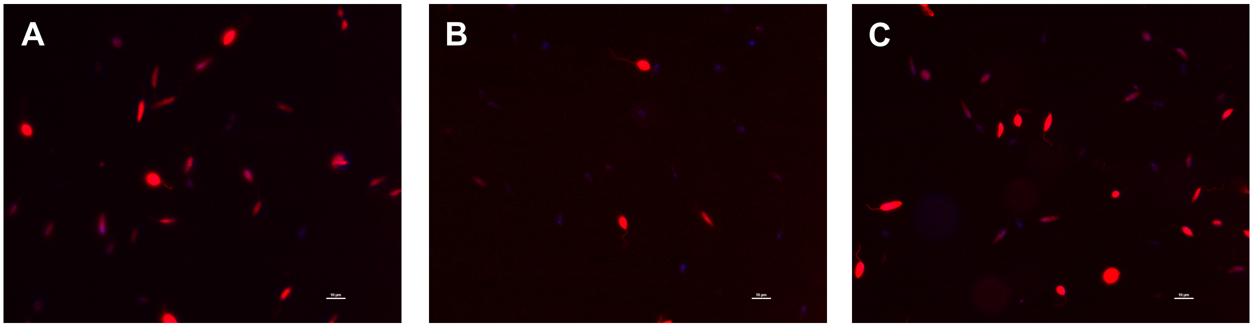

Figure S5. Detection of red fluorescent parasites by confocal microscopy. **A)** pXG-mCherry12 parasites were grown in Hygromycin-supplemented medium and the red fluorescence emission was detected. **B)** When pXG-mCherry12 parasites were cultured for 60 days in Hygromycin-free medium, the fluorescence intensity decreased. **C)** On the contrary, after pXG-mCherry12 cells were grown in HFM (60 days) and then in Hygromycin-supplemented medium, the fluorescence intensity was recovered.

References

1 Lee, C., Kim, J., Shin, S.G. and Hwang, S. (2006) Absolute and Relative QPCR Quantification of Plasmid Copy Number in *Escherichia coli*. *Journal of biotechnology*, **123**, 273–280. http://dx.doi.org/10.1016/j.jbiotec.2005.11.014.

2 Van den Bossche, J., Baardman, J. and de Winther, M.P.J. (2015) Metabolic Characterization of Polarized M1 and M2 Bone Marrow-Derived Macrophages Using Real-Time Extracellular Flux Analysis. *Journal of visualized experiments: JoVE*. http://dx.doi.org/10.3791/53424.

3 Calvo-Álvarez, E., Guerrero, N.A., Alvarez-Velilla, R., Prada, C.F., Requena, J.M., Punzón, C., Llamas, M.Á., Arévalo, F.J., Rivas, L., Fresno, M., Pérez-Pertejo, Y., Balaña-Fouce, R. and Reguera, R.M. (2012) Appraisal of a *Leishmania major* Strain Stably Expressing mCherry Fluorescent Protein for Both in Vitro and in Vivo Studies of Potential Drugs and Vaccine Against Cutaneous Leishmaniasis. Milon, G., Ed.,

PLoS Neglected Tropical Diseases, **6**, e1927. http://dx.doi.org/10.1371/journal.pntd.0001927.
